# *Solanum clarum* and *S. morelliforme* as Novel Model Species for Studies of Epiphytism

**DOI:** 10.3389/fpls.2016.00231

**Published:** 2016-02-29

**Authors:** Shelley H. Jansky, Jacob Roble, David M. Spooner

**Affiliations:** ^1^United States Department of Agriculture-Agricultural Research Service, Vegetable Crops Research UnitMadison, WI, USA; ^2^Department of Horticulture, University of Wisconsin-MadisonMadison, WI, USA

**Keywords:** epiphyte, mineral uptake, *Solanum clarum*, *Solanum morelliforme*

## Abstract

The natural history of epiphytic plant species has been extensively studied. However, little is known about the physiology and genetics of epiphytism. This is due to difficulties associated with growing epiphytic plants and the lack of tools for genomics studies and genetic manipulations. In this study, tubers were generated from 223 accessions of 42 wild potato *Solanum* species, including the epiphytic species *S. morelliforme* and its sister species *S. clarum*. Lyophilized samples were analyzed for 12 minerals using inductively coupled plasma optical emission spectrometry. Mineral levels in tubers of *S. morelliforme* and *S. clarum* were among the highest for 10 out of the 12 elements evaluated. These two wild potato relatives are native to southern Mexico and Central America and live as epiphytes or in epiphytic-like conditions. We propose the use of *S. morelliforme* and *S. clarum* as model organisms for the study of mineral uptake efficiency. They have a short life cycle, can be propagated vegetatively via tubers or cuttings, and can be easily grown in controlled environments. In addition, genome sequence data are available for potato. Transgenic manipulations and somatic fusions will allow the movement of genes from these epiphytes to cultivated potato.

## Introduction

Epiphytes encompass an unusual group of plant species that grow on other plants, typically in the crowns of trees, without parasitizing them. They are considered one of the most threatened plant groups (Mondragon et al., 2015). The 27,614 species of vascular epiphytes account for 9% of extant vascular plant diversity (Zotz, 2013). Vascular epiphytes range from ferns to flowering orchids and bromeliads. The biology and ecology of these unique plants has been extensively studied (reviewed by Mondragon et al., 2015). However, the physiology and genetics of epiphytism has received much less attention.

There are two main types of vascular epiphytes (Cardelús and Mack, 2010). The first acquires nutrients through organic debris that accumulates on the branches of host plants. This decaying organic debris, called crown humus, accumulates slowly over many years, forming a medium in which epiphytes, such as some fern species, can root and absorb nutrients (Jenik, 1973). Other epiphytes, such as bromeliads, obtain nutrients from the atmosphere through foliar feeding.

The main constraints on epiphytic growth and function are water acquisition, mineral procurement and utilization, and light exposure (Benzing, 1990; Laube and Zotz, 2003). Optimal growth requires the uptake of adequate levels of all essential minerals. The quality of the nutrient medium in the forest canopy can be highly variable and dependent on altitude, climate, humidity, and position above ground level. However, it is generally assumed that epiphytic habitats tend to be low in nutrients and sporadic in water supply (Laube and Zotz, 2003; Zotz, 2004; Zotz and Richter, 2006; Winkler and Zotz, 2008; Cardelús, 2009; Cardelús et al., 2009; Zotz and Asshoff, 2010).

Epiphytes have evolved to grow in low input environments. Adaptations to low mineral environments include slow growth rate, small stature, asexual reproduction, sexual reproduction with a minimum expenditure of non-recoverable mineral nutrients in seed and fruit production, resistance to mineral loss by leaching, tolerance of low mineral levels in living tissue, the capacity to substitute one element for another in metabolism, the ability to exploit mineral sources normally unavailable to higher plants, and the ability to absorb and sequester minerals in dilute solutions (Benzing, 1990; Schmidt and Zotz, 2002; Winkler and Zotz, 2008). The latter strategy is of most interest to scientists seeking to improve nutrient use efficiency in plants. Despite an abundance of research detailing the unique adaptations of epiphytes and their environments, the literature on mineral uptake is largely descriptive, and the genetic and physiological mechanisms of these processes are not well-understood (Luttge, 1989; Benzing, 1990; Zotz and Hietz, 2001; Rains et al., 2003; Zotz, 2004).

An improved understanding of the molecular basis of mineral uptake in epiphytes would contribute to many fields, including conservation biology, germplasm development, crop breeding, and plant physiology. This paper presents two wild potato (*Solanum* section *Petota*) relatives as model systems for the identification and characterization of genes responsible for nutrient acquisition and accumulation.

## Materials and methods

In October, 2007, true potato seed from 134 accessions (populations) of 42 wild *Solanum* species was obtained from the U.S. Potato Genebank (NRSP-6). Fifty seeds of each accession were sown in soilless potting mix (Pro-Mix™) and 3 weeks later, 15 seedlings per accession were transplanted into individual 5 cm pots. After another 3 weeks of growth, seedlings were transplanted into 10 cm pots. They were grown under high intensity (1000 w high pressure sodium) lights with an 18 h photoperiod. Day/night temperatures were 20C/16C. Plants were watered as needed, typically daily. Osmocote slow release fertilizer (19-6-12) was incorporated into the potting mix during transplanting. In January, photoperiod was reduced to 12 h to induce tuberization. Six weeks later, the trial was harvested and the largest tuber from each of the 15 plants in an accession was collected and all 15 tubers were placed in a paper bag. After 3 days at room temperature, tubers were immersed in liquid nitrogen and then placed in a −80°C freezer. Tubers were lyophilized and ground using a mortar and pestle. Tuber tissue from the 15 plants in each accession was combined for mineral analysis. For each sample, 500 mg of dried tuber tissue and 5 mL of concentrated nitric acid were added to a 50-mL Folin digestion tube. The mixture was heated to 120–130°C for 14–16 h and then treated with hydrogen peroxide. After digestion, the sample was diluted to 50 mL. This solution was analyzed for mineral content using inductively coupled plasma optical emission spectrometry (Model IRIS Advantage, Thermo Jarrell Ash, Waltham, MA). The trial was repeated in 2008 with 89 additional accessions.

## Results and discussion

This study was initiated as a survey of mineral uptake capacity in a geographically and taxonomically diverse set of wild *Solanum* species. However, after the mineral data were collected and species were compared, *S. morelliforme* and *S. clarum* stood out as exceptional for tuber mineral content. Tuber mineral levels, averaged by species, are presented in Table 1. Supplementary Table 1 provides maximum and Supplementary Table 2 provides minimum tuber mineral levels. At this point, and realizing that these species are epiphytes or found in epiphytic-like conditions, we began to consider the possibility that they could serve as model species for studies of mineral nutrient uptake.

**Table 1A:**
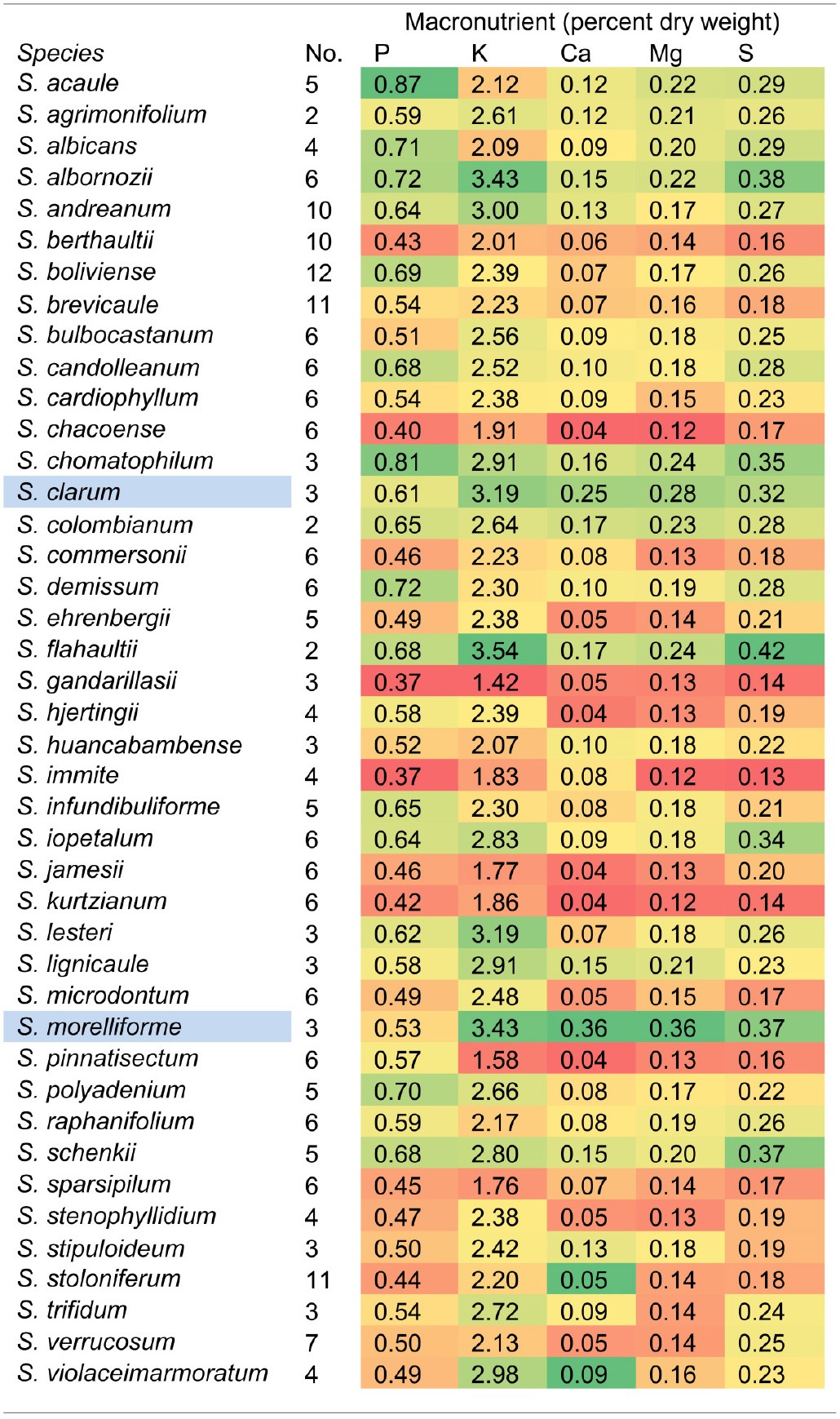
**Average tuber mineral levels of macronutrients in 42 wild ***Solanum*** species**.

*No. refers to the number of accessions sampled. Heat map shows higher concentrations in deeper shades of green and lower concentrations in deeper shades of red. Epiphytic species are highlighted in blue*.

**Table 1B:**
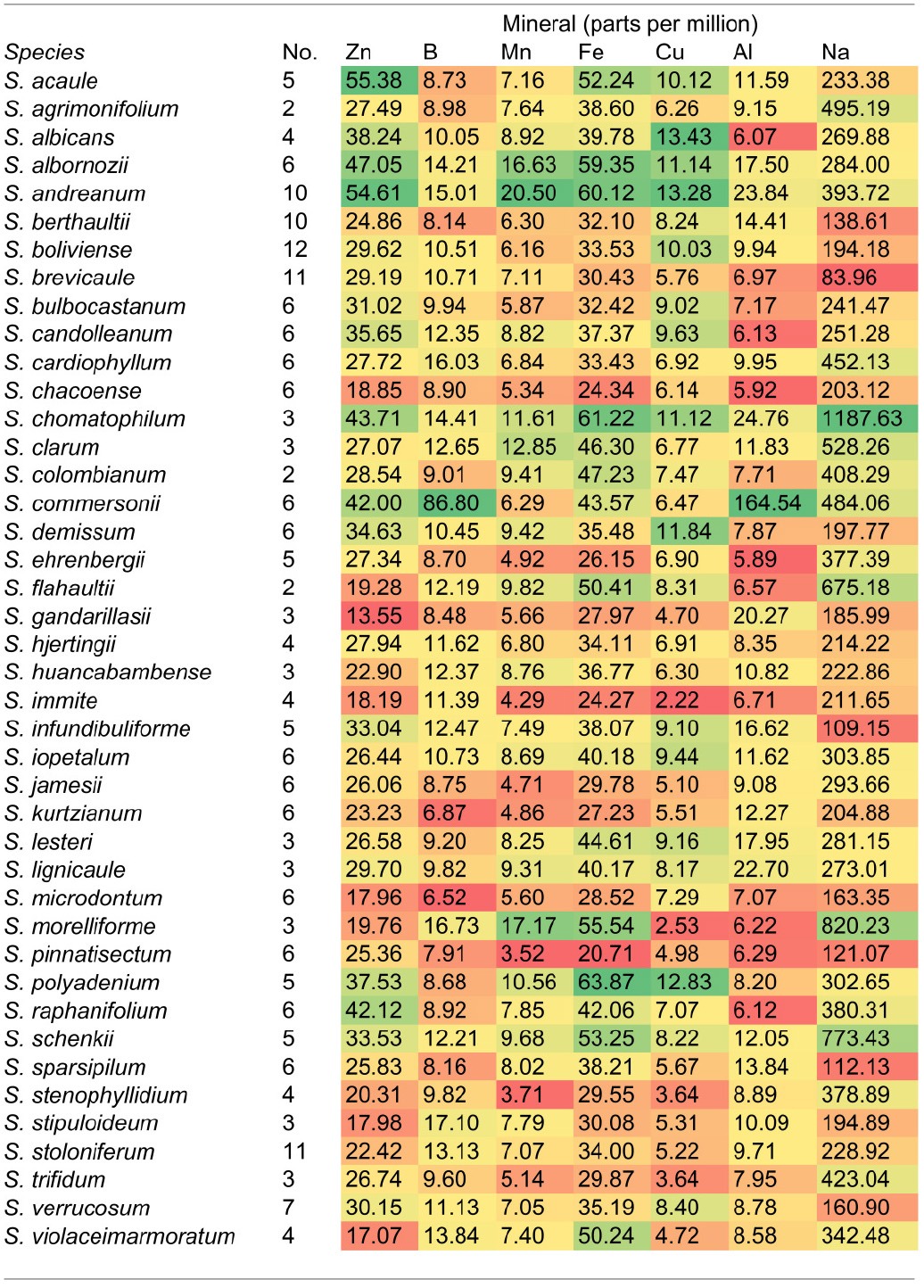
**Average tuber levels of minerals other than macronutrients in 42 wild ***Solanum*** species**.

*No. refers to the number of accessions sampled. Heat map shows higher concentrations in deeper shades of green and lower concentrations in deeper shades of red. Epiphytic species are highlighted in blue*.

**Figure 1:**
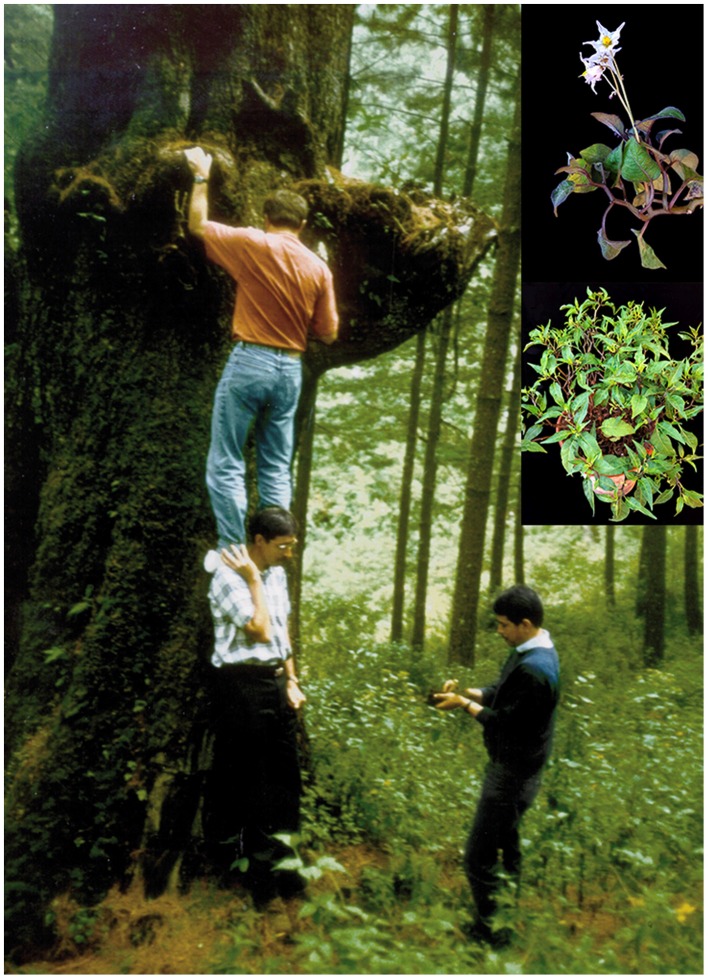
**Collecting epiphytic ***Solanum morelliforme*****. Inserts are photos of *S. clarum* (upper) and *S. morelliforme*.

*Solanum morelliforme* Bitter & Muench is a diploid (2*n* = 24), self-incompatible, epiphytic member of *Solanum* section *Petota*. It is widespread throughout central Mexico (southern Jalisco to Querétato and Veracruz), south to southern Honduras, growing from 1870 to 3050 m in elevation, flowering and fruiting from July through October. A strikingly disjunct (approximately 4000 km) population was recently discovered in Bolivia, representing the first record of this species in South America, and the first species in the section growing in both North and Central America and in South America (Simon et al., 2011). *Solanum morelliforme* is distinctive with its simple leaves, relatively small stature (stems 2–3 mm wide at base, 0.1–0.5 m tall), epiphytic habit, and is impossible to be confused with any other wild potato. *Solanum morelliforme* is the only epiphytic wild potato, growing on horizontal branches of mature *Arbutus* L., cyprus, elm, juniper, pine, or oak trees, often rooted in moss and organic litter (Spooner et al., 2004; Figure 1). Field studies in Mexico and Central America (Spooner et al., 1998, 2000) showed that it is difficult to find in previously documented localities that had been logged and reforested, suggesting that its range is being reduced by deforestation.

*Solanum morelliforme* is most similar morphologically to *S. clarum* Correll, its sister species (Spooner et al., 2004). *Solanum clarum* is distributed in southern extreme Mexico and Guatemala, from 2740 to 3800 m in elevation, flowering and fruiting from July through November. Like *S. morelliforme, S. clarum* is a diploid (2*n* = 24). Although not technically an epiphytic species, it occasionally grows in trees but more commonly in epiphytic-like conditions, in shade, in upland pine and fir forests, frequently associated with *Acaena elongata* L., *Alchemilla pectinata* H. B. K., or *Pernettya ciliata* (Schltdl. & Charn) (Spooner et al., 2004).

The three *S. clarum* accessions and one *S. morelliforme* accession were collected in Guatemala. The remaining two *S. morelliforme* accessions originate from Mexico. Passport data reveal that one of the *S. clarum* accessions and two of the *S. morelliforme* accessions were growing as epiphytes when they were collected. They have many characteristics of plants adapted to mineral-deficient environments including small stature, asexual reproduction via tubers, small fruits bearing few seeds, and infrequent sexual reproduction (Spooner et al., 2004).

The two species proposed as models in this paper differ in that *S. morelliforme* is typically found in trees, while *S. clarum* may be in trees or in the litter surrounding trees. Attempts have been made to distinguish among gradations in the proportion of plants of an epiphytic species that are found growing in trees (Zotz, 2013). However, the environment on a fallen tree or the mossy low branches of a tree is often very similar to that of both the moss-covered ground and an intact mature tree.

Because nutrient supply is low and irregular in crown humus, epiphytes must possess highly efficient mineral uptake and utilization mechanisms (Benzing and Renfrow, 1974). In addition, when provided with the opportunity, they may take up minerals in excess of current needs and store them for future use, a phenomenon called luxury consumption (Benzing and Renfrow, 1980; Chapin, 1980; Benzing, 2000). Storage organs, such as the potato tubers evaluated in this study, provide a natural mechanism for accumulating and storing mineral nutrients.

Phosphorus is often a limiting nutrient for many vascular epiphytes in tropical forests Epiphytic bromeliads have been shown to efficiently take up phosphorus and then store it for later use (Winkler and Zotz, 2008; Zotz and Asshoff, 2010). The two *Solanum* epiphyte and epiphytic-like species in this study were also found to have high phosphorus levels in storage organs, compared to a wide array of wild potato species (Table 1).

While this paper has focused on epiphytic relatives of potato, the tuber mineral survey revealed non-epiphytic species that may also be useful in mineral nutrition studies. *Solanum albornozii* and *S. flahaultii*, for example, were among the highest ranked species for several minerals. A large amount of phenotypic variation, and presumably genotypic variation, is common within accessions in wild potato (Bamberg et al., 1996; Douches et al., 2001; Jansky et al., 2006, 2008, 2009; Spooner et al., 2009; Chung et al., 2010; Cai et al., 2011). This is expected, considering the wide range of habitats in which wild *Solanum* species grow.

Anatomical features for mineral uptake, such as tanks in bromeliads and aerial roots in orchids are not found in *S. morelliforme* and *S. clarum*. In non-epiphytic potato, calcium uptake has been studied intensively, revealing two types of enhanced mineral uptake mechanisms based on physiological rather than anatomical adaptations (Bamberg et al., 1993, 1998). In one system, plants are able to take up adequate nutrients from a low nutrient environment. In the second system, plants accumulate high levels of calcium from an environment with moderate levels of the mineral. Nutrient efficient plants may possess one or both of these mechanisms (Bamberg et al., 1993, 1998). Mineral uptake mechanisms have not yet been characterized in epiphytic potato. However, it appears that they must rely on physiological rather than anatomical mineral uptake mechanisms to survive in nutrient-poor crown humus.

An ideal model epiphytic plant species would have a short life cycle and be capable of rapid and reliable asexual reproduction. Many epiphytic species require 10–20 years to reach sexual maturity (Mondragon et al., 2015). *Solanum clarum* and *S. morelliforme*, however, reach maturity in a matter of months. They are easily propagated asexually via stem-leaf cuttings or as tissue culture plantlets, have small space requirements (they grow readily in peat-based potting mix in small pots), and do not require high intensity light for growth. All these features make them useful model organisms for studying the biology of epiphytes.

The potato genome has been sequenced (The Potato Genome Sequencing Consortium, 2011) providing the opportunity to carry out gene discovery studies related to nutrient acquisition and storage in epiphytic relatives. The identification of the genes responsible for efficient nutrient uptake can be used to find orthologous sequences in other species. In addition, these genes may be transferred into cultivated potato. *Solanum morelliforme* and *S. clarum* are likely sexually incompatible with cultivated potato. However, genetic transformation in potato is straightforward and transgenic technology is well-established (Millam, 2009). Alternatively, somatic fusion protocols are in place and have been used to introgress the genomes of tertiary gene pool species into cultivated potato (Austin et al., 1985, 1988).

The U.S. Potato Genebank (NRSP-6) maintains 23 accessions of *S. morelliforme* and 14 accessions of *S. clarum*. This germplasm is freely available upon request to NRSP-6. Information about these accessions can be found on the USDA Germplasm Resources Information Network (www.ars-grin.gov).

## Author contributions

SJ generated the research material, carried out the mineral analyses, and supervised the writing of the manuscript. DS initiated the research project, determined the species and accessions to be analyzed and edited the manuscript. JR carried out the literature review and wrote the majority of the manuscript.

### Conflict of interest statement

The authors declare that the research was conducted in the absence of any commercial or financial relationships that could be construed as a potential conflict of interest.

## Supplementary material

Supplementary Table 1 **Maximum tuber mineral levels in 42 wild *Solanum* species**. No. refers to the number of accessions sampled. Epiphytic species are highlighted in blue.

Supplementary Table 2 **Minimum tuber mineral levels in 42 wild *Solanum* species**. No. refers to the number of accessions sampled. Epiphytic species are highlighted in blue.
